# Asymmetric dimethylarginine predicts decline of glucose tolerance in men with stable coronary artery disease: a 4.5-year follow-up study

**DOI:** 10.1186/1475-2840-12-64

**Published:** 2013-04-11

**Authors:** Andrzej Surdacki, Olga Kruszelnicka, Tomasz Rakowski, Aleksandra Jaźwińska-Kozuba, Jacek S Dubiel

**Affiliations:** 1 2nd Department of Cardiology, Faculty of Medicine, Jagiellonian University / University Hospital, 17 Kopernika Street, Cracow 31-501, Poland; 2 Department of Coronary Artery Disease, the John Paul II Hospital, 80 Prądnicka Street, Cracow 31-202, Poland; 3 Almed-Elektra Medical Center, 288 1-go Maja Street, Ruda Śląska 41-710, Poland

**Keywords:** ADMA, Coronary artery disease, Insulin resistance, New-onset type 2 diabetes, Pre-diabetes

## Abstract

**Background:**

Endothelial dysfunction, largely dependent on impaired nitric oxide bioavailability, has been reportedly associated with incident type 2 diabetes. Our aim was to test the hypothesis that asymmetric dimethylarginine (ADMA), an endogenous inhibitor of nitric oxide formation, might be linked to future deterioration in glucose tolerance in stable coronary artery disease (CAD).

**Methods:**

We studied 80 non-diabetic men (mean age 55 ± 11 years) with stable angina who underwent successful elective complex coronary angioplasty and were receiving a standard medication according to practice guidelines. Plasma ADMA and its structural isomer symmetric dimethylarginine (SDMA) were measured prior to coronary angiography. An estimate of insulin resistance by homeostasis model assessment (HOMA-IR index) was calculated from fasting insulin and glucose. Deterioration in glucose tolerance was defined as development of type 2 diabetes or progression from a normal glucose tolerance to impaired fasting glucose.

**Results:**

Over a median follow-up of 55 months 11 subjects developed type 2 diabetes and 13 progressed to impaired fasting glucose. Incident deterioration of glucose tolerance was associated with ADMA (hazard ratio [HR] per 1-SD increment 1.64 [95% CI: 1.14–2.35]; *P* = 0.007), log (HOMA-IR index) (HR = 1.60 [1.16–2.20]; *P* = 0.004) and body-mass index (HR = 1.44 [0.95–2.17]; *P* = 0.08) by univariate Cox regression. ADMA (HR = 1.65 [1.14–2.38]; p = 0.008) and log (HOMA-IR index) (HR = 1.55 [1.10–2.17]; *P* = 0.01) were multivariate predictors of a decline in glucose tolerance. ADMA and SDMA were unrelated to body-mass index, HOMA-IR index, insulin or glucose.

**Conclusions:**

ADMA predicts future deterioration of glucose tolerance independently of baseline insulin resistance in men with stable CAD. Whether this association reflects a contribution of endothelial dysfunction to accelerated decline of insulin sensitivity, or represents only an epiphenomenon accompanying pre-diabetes, remains to be elucidated. The observed relationship might contribute to the well-recognized ability of ADMA to predict cardiovascular outcome.

## Background

Endothelial dysfunction, a predecessor of atherosclerotic plaques and adverse cardiovascular (CV) events, is largely mediated by depressed bioavailability of nitric oxide (NO) [1]. Biochemical markers of endothelial activation [2-4] and impaired endothelium-dependent vasodilation [5] have also been identified as predictors of new-onset type 2 diabetes independently of the degree of insulin resistance (IR) [2,5], a major precursor of non-insulin-dependent diabetes [6-9] and coronary atherosclerosis progression [10,11].

The endogenous inhibitor of NO formation, asymmetric dimethylarginine (ADMA), is associated with endothelial dysfunction and adverse outcome predictor including all-cause mortality and CV events in patients with coronary artery disease (CAD) and in the general population [12]. Mittermayer et al. [13] identified elevated ADMA but not IR as an independent predictor for deterioration in glucose tolerance in women with a history of previous gestational diabetes mellitus. To the best of our knowledge, there are no other reports on the association between endogenous dimethylarginines and incident type 2 diabetes. In addition, there are conflicting data on relations between ADMA and the magnitude of IR in human studies [14-23].

Therefore, our aim was to test the hypothesis that plasma ADMA levels might be linked to a future decline of glucose tolerance in men with stable CAD.

## Methods

### Patients

We studied the previously characterized group of 80 non-diabetic men with stable angina who underwent successful elective complex coronary angioplasty with implantation of ≥1 bare-metal stent in our tertiary care center due to the presence of a diameter stenosis of ≥70% of ≥1 major epicardial artery segment [24,25]. Prior to the hospitalization, the patients were on low-dose aspirin, angiotensin-converting enzyme inhibitors (ACEI) and statins for ≥3 months and this medication was maintained after discharge and supplemented by clopidogrel following current practice guidelines (Tables 1 and 2). As described [24], exclusion criteria included diabetes (by either fasting glucose or postload glycemia during an oral glucose tolerance test (OGTT)), proteinuria, estimated glomerular filtration rate (eGFR) <30 mL/min per 1.73 m^2^ body-surface area, any surgery within past 6 months, acute coronary syndromes within past 3 months, depressed left ventricular ejection fraction (by ultrasound), heart failure, uncontrolled hypertension, overt extracoronary atherosclerosis, chronic coexistent diseases, infections within previous 2 months, relevant abnormalities in routine laboratory assays and any chronic non-CV medication. Out of 358 potentially eligible consecutive patients, 275 were eliminated by the exclusion criteria and 3 due to the discontinuation of ACEI or statins during the follow-up, as indicated previously [24,25].

**Table 1:** Baseline patients’ characteristics by incident deterioration in glucose tolerance

**Variable**	**Deterioration in glucose tolerance (*n*** **= 24)**	**No deterioration in glucose tolerance (*n*** **= 56)**	***P *value**
Age (years)	57 ± 11	54 ± 10	0.32
BMI (kg/m^2^)	28.1 ± 4.1	26.6 ± 3.4	0.11
Current smokers, *n* (%)	5 (21%)	15 (27%)	0.57
one-vessel/multivessel CAD, n (%)	5/19 (21/79%)	16/40 (29/71%)	0.47
Left ventricular ejection fraction (%)	71 ± 6	70 ± 5	0.49
Hypertension, *n* (%)	20 (83%)	42 (75%)	0.42
Mean blood pressure (mm Hg)	95 ± 8	96 ± 9	0.85
Estimated GFR (mL/min per 1.73 m^2^)	71 ± 10	68 ± 12	0.26
LDL cholesterol (mmol/L)	2.5 ± 0.8	2.6 ± 0.7	0.79
HDL cholesterol (mmol/L)	0.8 ± 0.3	0.9 ± 0.4	0.22
Triglycerides (mmol/L)	1.5 ± 0.8	1.3 ± 0.7	0.40
Hs-CRP (mg/L)	1.9 (0.6–9.1)	1.6 (0.5–8.6)	0.16^*^
Fasting glucose (mmol/L)	6.0 ± 0.8	5.7 ± 0.8	0.17
Fasting insulin (μU/ml)	13.5 (4.9–50.2)	12.4 (5.3–43.5)	0.04^*^
HOMA-IR index	3.50 (1.78–13.8)	3.06 (1.44–10.7)	0.03^*^
ADMA (μmol/L)	0.53 ± 0.14	0.46 ± 0.10	0.02^†^
SDMA (μmol/L)	0.64 ± 0.15	0.68 ± 0.13	0.27
L-arginine (μmol/L)	68 ± 19	67 ± 20	0.86
Drugs besides aspirin + ACEI + statin			
β-blockers, *n* (%)	17 (71%)	45 (80%)	0.35
Long-acting nitrates, *n* (%)	23 (96%)	50 (89%)	0.34
Calcium channel blockers, *n* (%)	8 (33%)	15 (27%)	0.55

Data are shown as mean ± SD, median (range) or *n* (%).

^*^ By Student’s *t*-test for log-transformed data.

^†^ By Welch’s *t*-test for unequal variances.

Abbreviations: ACEI, angiotensin-converting enzyme inhibitors; ADMA, asymmetric dimethylarginine; BMI, body-mass index; CAD, coronary artery disease; GFR, glomerular filtration rate; HDL, high-density lipoproteins; HOMA-IR, homeostasis model assessment for insulin resistance; Hs-CRP, high-sensitivity C-reactive protein; LDL, low-density lipoproteins; SDMA, symmetric dimethylarginine.

**Table 2:** Cardiovascular drugs used during the follow-up period^*^

**Drug class, *n *(%)**	**Deterioration in glucose tolerance (*n*** **= 24)**	**No deterioration in glucose tolerance (*n*** **= 56)**	***P *value**
Diuretics	6 (25%)	15 (27%)	0.87
β-blockers	19 (79%)	50 (89%)	0.23
α_1_-blockers	5 (21%)	11 (20%)	0.91
Calcium channel blockers	10 (42%)	19 (34%)	0.51
Angiotensin receptor antagonists	2 (8%)	3 (5%)	0.61
Long-acting nitrates	9 (38%)	20 (36%)	0.88

^*^ Besides low-dose aspirin, angiotensin-converting enzyme inhibitors, statins and clopidogrel.

The study was conducted in compliance with the Declaration of Helsinki; the ethical committee of our university had approved the protocol and informed consent was obtained from each patient.

### Biochemical assays

Blood samples for extended biochemical assays were drawn from an antecubital vein after an overnight fast at routine blood sampling 0–2 days before the planned angiography. Plasma (collected from ethylenediaminetetraacetic acid-anticoagulated blood) and serum were separated and stored at −70°C until assayed [24,25]. Routine analyses included complete blood count, lipids, glucose, and creatinine using standardized laboratory techniques. We calculated eGFR by the simplified formula developed by the Modification of Diet in Renal Disease Study Group [26].

In addition, high-sensitivity C-reactive protein (hs-CRP) and homocysteine were measured by chemiluminescent immunoassay systems (Immulite 1000 and Immulite 2000, DPC, Flanders, NJ, USA). A measure of IR by the homeostasis model assessment (HOMA-IR index) was calculated as a product of fasting plasma levels of insulin (μU/mL) and glucose (mmol/L) divided by 22.5 [27].

Plasma ADMA and SDMA were determined by means of commercially available enzyme-linked immunosorbent assays (ELISA) (DLD Diagnostika GmbH., Hamburg, Germany), previously validated against high-performance liquid chromatography coupled to mass spectrometry, the golden standard for the determination of ADMA levels [28]. According to the manufacturer, the lower detection limit was 0.05 μmol/L for both analytes and intra-assay and inter-assay coefficients of variation averaged 7.5 and 10.3% (ADMA) and 6.1 and 9.8% (SDMA), respectively [24]. Cross-reactivity with L-arginine and other methylarginines was <0.01–0.02% (SDMA or ADMA vs. L-arginine), 0.44 − 1.2% (ADMA vs. SDMA), and 0.7–1.0% (SDMA or ADMA vs. *N*^*G*^-monomethyl-L-arginine). L-arginine was also measured by ELISA (DLD Diagnostika GmbH., Hamburg, Germany); the lower detection limit was 3.0 μmol/L and intra-assay and inter-assay coefficients of variation averaged 3.6 and 8.3%, respectively, and cross-reactivity 0.01% (ADMA) and 0.68% (SDMA) as described by the manufacturer.

### Collection of follow-up data

After discharge from our center the patients were contacted directly at routine control visits in our outpatient clinic or by telephone for the occurrence of deterioration of glucose tolerance that was then confirmed by the review of hospital charts or other medical records and the date of diagnosis was ascertained. The personnel involved in the collection of follow-up data were unaware of patients’ laboratory background.

In agreement with the 2003 recommendations of the American Diabetes Association [29], diabetes was diagnosed in the presence of a fasting venous plasma glucose ≥7 mmol/L or casual plasma glucose ≥11.1 mmol/L in a patient with classic symptoms of hyperglycemia, a fasting venous plasma glucose ≥7 mmol/L confirmed by repeat testing if the diagnosis was not clear on clinical grounds, or introduction of insulin or an oral hypoglycemic drug. Impaired fasting glucose (IFG) and normal glucose tolerance were defined as fasting glucose of 5.6–6.9 mmol/L and <5.6 mmol/L, respectively [29]. As results of an OGTT during the follow-up were available only in 20 men, exclusively IFG was classified as pre-diabetes to avoid a bias caused by the lack of repeated OGTT over time. Deterioration in glucose tolerance was defined as progression from a normal glucose tolerance to IFG or development of type 2 diabetes irrespective of baseline glucose tolerance status. Exclusively a documented decline in glucose tolerance in stable clinical conditions (e.g., not during a hospitalization due to an acute coronary syndrome) was classified as an outcome event.

### Statistical analysis

Data are presented as means ± SD for continuous variables with normal distribution, medians and range or interquartile range for not normally distributed parameters, and numbers (proportions) for categorical variables. The accordance with a normal distribution was confirmed by the Kolmogorov-Smirnov test and uniformity of variance by Levene’s test. Natural logarithmic transformation was applied for fasting insulin, HOMA-IR index and hs-CRP to get a normal distribution. Additionally, log (HOMA-IR index) was entered into further analyses due to its better ability to predict the glucose clamp-derived reference measure of insulin sensitivity compared to untransformed values exhibiting a right-skewed distribution [30].

Intergroup comparisons of normally distributed parameters between patients with a stable and deteriorated glucose tolerance were performed by the 2-sided Student’s *t*-test or Welch’s *t*-test in case of the lack of homogeneity of variance. Proportions were compared by the chi-squared test. Bivariate relations between continuous data were assessed by Pearson’s correlation coefficients (*r*).

Predictors of the risk of a decline in glucose tolerance (outcome event) were estimated by event-free survival analysis with the last contact with the patient treated as a censored event if no change in glucose tolerance status was recorded. We have not applied a competing risk modeling approach due to a low number of deaths (*n* = 3) in our study group. Kaplan-Meier event-free survival curves were compared by the log-rank test with ADMA levels categorized according to the median (0.45 μmol/L) because a preliminary analysis revealed no significant differences between respective curves for the first and second quartile of ADMA concentrations. Cox proportional hazards regression was performed after validating the proportionality assumption, i.e., the lack of a significant effect of the interaction term between each covariate and time, when added into regression models. Interaction within pairs of covariates were estimated by calculating the significance for respective interaction terms. Independent determinants of deterioration in glucose tolerance were identified by multivariate Cox method including only covariates for which the *P* value at a univariate analysis did not exceed 0.15. Due to the collinearity between log-transformed values of insulinemia and HOMA-IR index (*r* = 0.96, *P* < 0.0001), exclusively the latter was entered into the Cox regression models. Mean standardized hazard ratios (HR) (per 1-SD increments in predictor variables), their 95% confidence intervals (CI) and associated *P* values were shown. Analyses were performed using STATISTICA (data analysis software system, version 10.0.1011.0; StatSoft, Inc., Tulsa, OK, USA). A *P* value <0.05 was inferred significant.

## Results

Over a median follow-up of 55 months (interquartile range 45–60 months) 11 subjects developed diabetes and further 13 progressed to IFG from a normal glucose tolerance. Compared to the patients with unchanged glucose tolerance status, those with deteriorated glucose tolerance exhibited significantly higher fasting insulin levels, HOMA-IR index and ADMA concentrations at baseline as well as insignificant tendencies towards a higher body-mass index (BMI), fasting glucose and hs-CRP (Table 1). There were no significant intergroup differences in L-arginine or SDMA levels (Table 1). CV drugs used during the follow-up period were similar among the both groups (Table 2).

Pooling both groups together, SDMA correlated to eGFR (*r* = −0.66, *P* < 0.001) and BMI to log-transformed values of HOMA-IR index (*r* = 0.37, *P* = 0.001) and of fasting insulin (*r* = 0.30, *P* = 0.007). Except for an insignificant tendency to a negative correlation between SDMA and log (HOMA-IR index) (*r* = −0.18, *P* = 0.12), plasma dimethylarginines were unrelated to BMI, HOMA-IR index, fasting insulin or glucose levels (*P* > 0.3).

**Figure 1:**
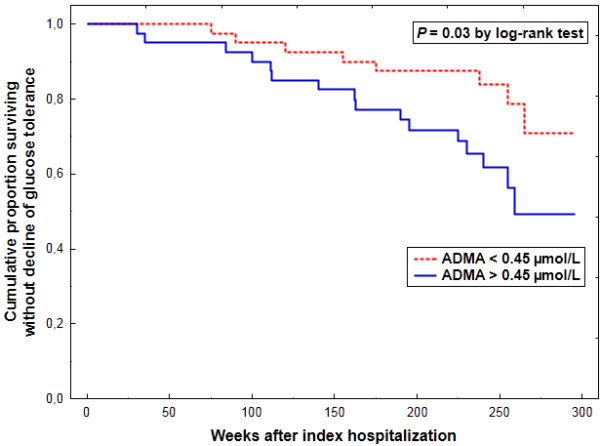
Cumulative proportion survival without deterioration in glucose tolerance according to baseline plasma asymmetric dimethylarginine (ADMA).

A depressed event-free survival was observed in the presence of an over-median ADMA level (*P =* 0.03 by the log-rank test) (Figure 1). Kaplan-Meier curves data have been shown in detail [see Additional file 1]. According to a univariate Cox regression, ADMA (HR = 1.64 [95% CI: 1.14–2.35]; *P* = 0.007) and log (HOMA-IR index) (HR = 1.60 [1.16–2.20]; *P* = 0.004) were associated with a significantly higher risk of deterioration in glucose tolerance, whereas a weaker respective relationship was found for BMI (HR = 1.44 [0.95–2.17]; *P* = 0.08). Neither SDMA (*P* = 0.4) nor L-arginine (*P* = 0.9) was related to the risk of progressive impairment of glucose tolerance. By a multivariate approach, ADMA and log (HOMA-IR index) were the only significant independent predictors of a decline in glucose tolerance (Table 3).

**Table 3:** Multivariate Cox regression analysis of the risk of incident decline of glucose tolerance

**Variable**	**SD**	Multivariable-adjusted^* ^**HR per 1-SD increment**		
		**Mean HR**	**95% CI**	***P *value**
ADMA	0.115 μmol/L	l.65	1.14–2.38	0.008
log (HOMA-IR index)	0.325	1.55	1.10–2.17	0.01
BMI	3.60 kg/m^2^	1.12	0.72–1.74	0.62

^*^ Adjusted for the remaining covariates listed in the Table.

Abbreviations: CI, confidence interval; SD, standard deviation; HR, hazard ratio; other abbreviations as in Table 1.

## Discussion

Our salient finding was an association between higher ADMA and future deterioration of glucose tolerance, irrespective of the degree of baseline IR, in men with stable CAD over an about 4.5-year follow-up.

### Endothelial dysfunction as a precursor of type 2 diabetes in previous clinical reports

Our results extend previous reports suggestive of an association between incident type 2 diabetes and biochemical indices of endothelial activation [2-4] or vasodilatory endothelial dysfunction [5], which retained significance upon multivariate adjustment including the HOMA-IR index [5] or fasting insulinemia [2], surrogate markers of IR. This association was interpreted as a possible consequence of an impaired insulin ability to stimulate blood flow via the L-arginine–NO pathway in the skeletal muscle and adipose tissue due to endothelial dysfunction at the arteriolar and capillary level with dysfunctional endothelium as an underlying cause of IR [2,3,31]. That adjustment for baseline insulin sensitivity (derived from the frequently sampled intravenous glucose tolerance test) only slightly attenuated the relationship between CV risk factors and a 5-year cumulative incidence of type 2 diabetes in 872 participants of the Insulin Resistance Atherosclerosis Study [32], is also in keeping with the predominant role of endothelial dysfunction with regard to the risk of developing diabetes. Given anti-inflammatory properties of the normal endothelium, this concept is also concordant with a joint synergistic contribution of higher hs-CRP and peripheral microvascular endothelial dysfunction, but not IR, to the risk of new-onset type 2 diabetes [5]. Admittedly, we observed no significant association between deteriorating glucose tolerance and hs-CRP in contrast to some previous reports [3,5,33]. Nevertheless, those studies were dealing with mainly untreated subjects, whereas at the time of blood collection our patients were chronically receiving statins, known to decrease CRP levels [34] but not ADMA [35], which might have obscured the discussed relationship. An analogous mechanism, in addition to the ability of ACEI, administered to all our subjects, to lower ADMA concentrations [36], could also be responsible for the lack of correlation between ADMA and hs-CRP. It is noteworthy that ADMA and hs-CRP were positively interrelated in the participants of the Ludwigshafen Risk and Cardiovascular Health study [37] but not the Athero*Gene* study [38], both of which were focused on CAD subjects presenting mainly stable angina.

### Associations between the L-arginine–NO–ADMA pathway and insulin signaling

Sydow et al. [39] reported enhanced whole-body insulin sensitivity in transgenic mice overexpressing dimethylarginine dimethylaminohydrolase (DDAH), an enzyme responsible for the predominant pathway of ADMA degradation [40]. Moreover, a more effective insulin signaling in the liver and a trend for increased baseline glucose uptake in an isolated fast-twitch muscle were observed. Additionally, in wild-type animals low ADMA levels (2 μmol/L) impaired insulin-mediated glucose uptake and incorporation of glucose into glycogen in an isolated fast-twitch muscle but not a slow-twitch muscle [39]. Furthermore, a functional variant of the DDAH-2 gene, linked to depressed expression of the isoform of DDAH which predominates in the vascular endothelium [40], was associated with a higher IR in 1,485 non-diabetic subjects of European ancestry, out of whom 527 had insulin sensitivity assessed as insulin-stimulated glucose disposal rate during the last hour of a hyperinsulinemic euglycemic clamp, the golden standard method [22]. According to Baron and colleagues [41], a superimposed intrafemoral artery infusion of *N*^*G*^-monomethyl-L-arginine during hyperinsulinemic euglycemic clamps not only abolished the insulin-dependent NO-mediated vasodilation [42,43] but also reduced leg glucose uptake. Thus, besides impaired hepatic insulin signaling, the ADMA-mediated inhibition of glucose uptake by the skeletal muscles – in part dependent on the blockade of vascular endothelial-type NO synthase (eNOS) and consequently blunted muscular perfusion under hyperinsulinemic conditions [41-43] – might have contributed to the relations between DDAH-2 polymorphism and diabetes incidence or IR [22], providing also a mechanistic explanation for our findings.

Additionally, a modulation of neuronal-type NO synthase (nNOS), whose variant is expressed predominantly in fast-twitch skeletal muscle fibers [44], might be responsible for the association between IR and the L-arginine–NO pathway. It is noteworthy that although both nNOS-knockout and eNOS-deficient mice were insulin-resistant compared to wild-type animals, the former exhibited IR exclusively at the level of peripheral tissues, whereas in the latter IR was present also in the liver [45]. Furthermore, a polymorphism of the gene encoding DDAH-1, an isoform of DDAH tending to accompany nNOS [40], was associated with both a decreased risk of type 2 diabetes and a tendency towards a lower HOMA-IR index in 814 Taiwanese subjects [46].

### ADMA levels versus the magnitude of IR in clinical conditions

Although the presented experimental and genetic data suggest reciprocal and stimulatory interactions between the L-arginine-NO pathway and metabolic sensitivity to insulin [22,39,41,45,46], there are conflicting reports on the relationship between ADMA and IR in clinical conditions [13-23]. Intriguingly, we detected no correlation between ADMA and the HOMA-IR index, in accordance with the data by Mittermayer et al. [13] who described an independent relationship between higher ADMA levels 14–16 weeks after delivery with future deterioration of glucose tolerance in 18 out of 77 women with previous gestational diabetes mellitus who progressed to impaired glucose tolerance in 9 and developed new-onset type 2 diabetes in further 9 cases after a median follow-up of almost 3 years. The lack of association between ADMA and the magnitude of IR was also observed by other authors, including ourselves [14,18-20], in contrast to studies reporting a positive relationship between the respective parameters [15-17,21,23]. Admittedly, the absence of positive correlations between ADMA and the HOMA-IR index or BMI in our subjects – in disagreement with a large study [23] – might have resulted from a relatively low inter-individual variability of ADMA values. Nevertheless, there are also inconsistent data on the effects of rosiglitazone, an insulin-sensitizing agent, on ADMA levels [15,47].

Ferrannini et al. [9] demonstrated that deterioration of glucose tolerance over time was associated with longitudinal decreases in both insulin sensitivity and β-cell sensitivity to glucose. Thus, given IR as the primary determinant of future development of type 2 diabetes [6-9], the present study suggests that endothelial dysfunction and higher ADMA might rather predispose to further potentiation of IR without an unequivocal association with IR at baseline in cross-sectional analyses [13-23]. Additionally, we cannot exclude the possibility that ADMA might progressively impair β-cell function, another contributor to the development of type 2 diabetes [6-9].

### Mechanistic considerations

Admittedly, a correlation does not imply a cause-and effect relationship and elevated ADMA might be only an epiphenomenon accompanying some abnormalities in the pre-diabetic state. These abnormalities can include higher glucose levels, a well-recognized cause of elevated ADMA via depressed DDAH activity due to a higher intracellular oxidative stress [48]. Additionally, the hyperglycemia-induced depression of DDAH activity [48] might counteract the hyperinsulinemia-stimulated uptake of ADMA into the cells via an augmented expression of cationic amino acid transporters (CAT) [49]. A complex interplay between these opposite effects of glucose and insulin on plasma ADMA [50] might be responsible for the previously mentioned discordant reports on the relationship between IR and ADMA [13-23]. Moreover, the same opposing regulatory mechanisms could also explain negative correlations between the HOMA-IR index and the SDMA/ADMA ratio [19] or SDMA [20] in Caucasians, an insignificant tendency to which was also found in our study group. Accordingly, the hyperinsulinemia-stimulated CAT-dependent uptake of ADMA and SDMA into the cells, probably within the liver and the kidney (both referred to as a “sink” for circulating ADMA [50]) – might hypothetically be counteracted – for ADMA but not SDMA – by the hyperglycemia-induced depression of DDAH activity [48]. In agreement with this concept, insulin-resistant subjects are prone to hyperinsulinemia and hyperglycemia and Eid et al. [51] reported a fall in ADMA during acute euglycemic hyperinsulinemia in humans. It is noteworthy that these two apparently contrary effects of insulin and glucose in terms of extracellular ADMA are likely to converge into increased intracellular ADMA levels [50]. Thus, the lack of relationship between plasma ADMA and HOMA-IR index, as in the present study, does not exclude the hypothetical continuous modulation of insulin signaling by intracellular ADMA.

Another interfering factor could be hepatic ability to clear plasma from endogenous dimethylarginines, responsible for about 30% and 20% of the combined hepatic and renal uptake of ADMA and SDMA in humans, respectively [52]. DDAH-1 expression was reduced during diabetogenesis in the liver, kidney and adipose tissue of mice with a point mutation in leptin receptor, an experimental motel of type 2 diabetes [53]. However, whether similar abnormalities occur in human pre-diabetic subjects, is as unclear as their speculative relevance for circulating ADMA levels or the risk of developing type 2 diabetes.

### Clinical implications

That our CAD subjects were receiving a standard therapeutic regimen in agreement with current guidelines, may point into a potential relevance of our results for the clinical reality. As progressive impairment of glucose tolerance is associated with a continuous rise in CV risk until its highest values in overt diabetes [54], the observed relation between elevated ADMA and a decline in glucose tolerance might contribute to the detrimental effect of ADMA on CV outcome [12,37,38]. Furthermore, Rijkelijkhuizen et al. [55] suggested conversion to type 2 diabetes as a basis of an excessive future CV mortality in Hoorn study participants exhibiting IFG. Therefore, besides the well-recognized link between ADMA and endothelial dysfunction [56], our finding might add to novel putative mechanisms of detrimental ADMA effects, e.g. its association with elevated arterial stiffness [57] or increased plasma viscosity [58] described in pre-diabetes and dyslipidemia, respectively. It is noteworthy that the correlation between ADMA and arterial stiffness in pre-diabetes was additive to an independent effect of low-density lipoproteins cholesterol [57]. Thus, excessive ADMA accumulation not only accompanies atherosclerotic risk factors, such as hypercholesterolemia [35] and essential hypertension [14], but also appears to amplify coexistent abnormalities.

Additionally, it is noteworthy that ACEI, angiotensin receptor blockers and statins – despite improving NO bioavailability – exert opposite effects on the risk of new-onset type 2 diabetes [59,60]. As ACEI and angiotensin receptor antagonists are able to lower ADMA levels [36] in contrast to the majority of reports on statins [35,40], our observation could provide a possible rationale for differential effects of these drug classes on the risk of developing type 2 diabetes.

### Study limitations

First, the lack of repeated determinations of ADMA, SDMA and IR over time constrains mechanistic conclusions based on a single measurement of these parameters in a relatively small number of study participants. Additionally, relatively high coefficients of variation of the ELISA assays for ADMA and SDMA pose another limitation. Second, we did not assess lifestyle factors or longitudinal changes in body weight, known to affect the risk of type 2 diabetes [9]. Third, in our retrospective analysis exclusively IFG was classified as pre-diabetes to avoid a bias resulting from the fact that results of an OGTT during the follow-up were available only in a minority of the study subjects. Fourth, although no significant intergroup differences in the use of various CV drug classes during the follow-up period were detected, the type of medication was determined exclusively by the analysis of an available medical documentation. Finally, IR was quantified on the basis of an indirect measure, i.e., the log-transformed HOMA-IR index. Nevertheless, this parameter not only exhibited a close correlation with the glucose clamp-derived insulin sensitivity index [30], but it also was one of the best predictors of incident type 2 diabetes among surrogate estimates of IR based on assays of fasting insulin and glucose [8].

## Conclusions

Our results suggest that higher plasma ADMA levels are associated with future deterioration of glucose tolerance over an about 4.5-year follow-up independently of the magnitude of baseline IR in men with stable CAD. Whether this association reflects a contribution of endothelial dysfunction to accelerated decline in insulin sensitivity, or represents only an epiphenomenon accompanying pre-diabetes, remains to be elucidated. The observed relationship might contribute to the well-recognized ability of ADMA to predict CV outcome.

## Abbreviations

ACEI: Angiotensin-converting enzyme inhibitors; ADMA: Asymmetric dimethylarginine; BMI: Body-mass index; CAD: Coronary artery disease; CAT: Cationic amino acid transporters; CI: Confidence interval; CV: Cardiovascular; DDAH: Dimethylarginine dimethylaminohydrolase; eGFR: Estimated glomerular filtration rate; eNOS: Endothelial-type nitric oxide synthase; HDL: High-density lipoproteins; HOMA-IR: Homeostasis model assessment for insulin resistance; HR: Hazard ratio; hs-CRP: High-sensitivity C-reactive protein; IFG: Impaired fasting glucose; IR: Insulin resistance; LDL: Low-density lipoproteins; nNOS: Neuronal-type nitric oxide synthase; NO: Nitric oxide; OGTT: Oral glucose tolerance test; SDMA: Symmetric dimethylarginine.

## Competing interests

The authors declare that they have no competing interests.

## Authors’ contributions

AS and OK conceived and designed the study, collected and analyzed data, and wrote the manuscript. AS and OK contributed equally to this work and are shared first authors. TR and AJK assisted with data collection and analysis and contributed to discussion. JSD contributed to the study design and discussion, and supervised the study. All authors read, critically revised and approved the final manuscript.

## Supplementary Material

Additional file 1 Kaplan-Meier curves data are shown as Additional file 1 entitled “Kaplan-Meier_curves_data.xls” including three columns: “time” (weeks after index hospitalization), “outcome” (0 = censored event; 1 = outcome event) and “ADMA > 0.45 μmol/L” (0 = a below-median ADMA level; 1 = an over-median ADMA level). Click here for file
